# Correlation of Frontal Atrophy and CSF Tau Levels With Neuropsychiatric Symptoms in Patients With Cognitive Impairment: A Memory Clinic Experience

**DOI:** 10.3389/fnagi.2021.595758

**Published:** 2021-03-05

**Authors:** Matteo Cotta Ramusino, Giulia Perini, Gloria Vaghi, Beatrice Dal Fabbro, Marco Capelli, Marta Picascia, Diego Franciotta, Lisa Farina, Elena Ballante, Alfredo Costa

**Affiliations:** ^1^Unit of Behavioral Neurology, IRCCS Mondino Foundation, Pavia, Italy; ^2^Department of Brain and Behavioral Sciences, University of Pavia, Pavia, Italy; ^3^Laboratory of Neuropsychology, IRCCS Mondino Foundation, Pavia, Italy; ^4^Laboratory of Neuroimmunology, IRCCS Mondino Foundation, Pavia, Italy; ^5^Neuroradiology Unit, IRCCS Mondino Foundation, Pavia, Italy; ^6^BioData Science Center, IRCCS Mondino Foundation, Pavia, Italy; ^7^Department of Mathematics, University of Pavia, Pavia, Italy

**Keywords:** behavioral and psychological symptoms of dementia, neuropsychiatric inventory, CSF biomarkers, atrophy visual rating scales, cognitive impairment

## Abstract

**Background**: Behavioral and psychological symptoms of dementia (BPSD) are a distressful condition. We aimed to investigate the BPSD distribution in subjects with cognitive impairment, and the potential correlations between BPSD and neurodegeneration in terms of cerebrospinal fluid (CSF) tau and brain atrophy.

**Methods**: One-hundred patients with mild cognitive impairment (MCI) or dementia (Alzheimer’s disease, AD; Lewy-body disease, LBD; frontotemporal dementia, FTD; vascular dementia, VD) underwent a complete diagnostic workup, including 3T-MRI and/or CT and CSF. Cortical atrophy was assessed with medial temporal atrophy (MTA), posterior atrophy (PA), and global cortical atrophy-frontal lobe (GCA-F) scales. BPSD were rated using the Neuropsychiatric Inventory (NPI), and BPSD clusters were defined according to the European Alzheimer Disease Consortium.

**Results**: Delusions, hallucinations, and psychosis cluster were differently distributed among the diagnostic groups (*p* < 0.05, *p* < 0.001, and *p* < 0.05), with LBD patients showing higher scores for hallucinations (vs. MCI, *p* < 0.001, and AD, *p* < 0.05) and psychosis cluster (vs. MCI, *p* < 0.05). In primary dementias, we found a negative correlation between NPI total score and tau levels (*p* = 0.08), confirmed by beta regression (*p* < 0.01), while a positive non-significant relationship was observed in MCI. Higher GCA-F scores were associated with delusions and apathy (*p* < 0.05, on both hemispheres) and hallucinations (left: *p* < 0.01, right: *p* < 0.05). GCA-F scores were positively correlated with psychosis cluster (right: *p* < 0.05), and agitation/aggression (left: *p* < 0.05). Conversely, nighttime disturbances were positively correlated with both GCA-F and MTA scores (left: *p* < 0.01; right: *p* < 0.05).

**Conclusion**: Our results suggest that psychotic symptoms are significantly more represented in LBD patients and that CSF tau and frontal atrophy are associated with the occurrence and severity of BPSD in clinical practice. Longitudinal studies are however required to ascertain their actual predictive value.

## Introduction

Dementia is a pathological condition with a strong impact on the quality of life of both patients and caregivers. In 2019 the World Health Organization estimated the global prevalence of dementia at around 50 million, with a trend to triple by 2050 (World Health Organization, 2019). The symptomatologic core of dementia consists of cognitive decline with a significant functional disability in daily life and progressive evolution (McKhann et al., 1984). Nonetheless, behavioral and psychological symptoms (BPSD) are a common feature affecting 98% of individuals with dementia (Phan et al., 2019), even in the early stages of disease (Di Iulio et al., 2010; Spalletta et al., 2012). These symptoms are usually referred to by the caregiver as the most critical and distressful aspects of the disease (McKeith and Cummings, 2005), and appear to be related to elevated disability, institutionalization, and death (Scarmeas et al., 2005). A recent study on a cohort of 181 subjects from the Alzheimer’s Disease Neuroimaging Study (ADNI) confirmed an inverse correlation between BPSD and cognitive/functional outcomes in patients with Alzheimer’s disease (AD), suggesting the importance of their early recognition to improve the disease management (Poulin et al., 2017). Confirming this, patients with amnestic mild cognitive impairment (MCI) and BPSD (in particular, apathy) had an almost sevenfold risk of AD progression (Palmer et al., 2010). Also, some BPSD display a good sensitivity and specificity, such as to have been included among the core or support diagnostic criteria of specific forms of dementia. This is the case of visual hallucinations and REM behavioral disorders in Lewy bodies dementia (LBD; McKeith et al., 2017), and disinhibition, apathy, aberrant motor behavior, and eating disorders in frontotemporal dementia (FTD; Rascovsky et al., 2011). It is thus essential, both for diagnosis and for management, to recognize these symptoms timely and track their evolution during the disease.

A possible link between BPSD patterns and profiles of cerebrospinal fluid (CSF) biomarkers has been often suggested, but conclusive data are still missing. In cognitively normal older adults, higher values of tau/Aβ42 were found to predict longitudinally greater increases of negative emotions, such as anxiety and depression (Babulal et al., 2016). In subjects with mild cognitive impairment (MCI), Ramakers and coll. observed a relationship between high *t*-tau levels and anxiety, and between low Aβ42 levels and agitation, irritability, and anxiety (Ramakers et al., 2013). Similarly, low Aβ42 levels were found to correlate with depression in another MCI population (Gonzales et al., 2018). In AD patients with mild-to-moderate dementia, high *t*-tau and *p*-tau levels were variously associated with different BPSD, in particular agitation and apathy (Skogseth et al., 2008; Bloniecki et al., 2014), and an inverse correlation between Aβ42 and aggressiveness was observed (Engelborghs et al., 2006).

A second relevant investigation field, based on neuroimaging, is the one focused on the research of possible links between morphological and functional cortical abnormalities and BPSD. Visual rating scales are a widespread and easy-to-use tool to assess atrophy, for both clinical and research purposes (Scheltens et al., 1992; Koedam et al., 2011; Van der Flier and Scheltens, 2018). In this regard, very little is known on the correlation among visual rating scales and BPSD: in AD, high medial temporal atrophy (MTA) scores were significantly associated with apathy and disinhibition (Garcia-Alberca et al., 2019), while posterior atrophy (PA) on the right hemisphere was associated to agitation and aggression (Hsu et al., 2015). Previous neuroimaging studies, assessing morphological, perfusion, and metabolic brain changes in AD patients, found that BPSD, such as delusions, apathy, and depression, were particularly associated with a frontal region involvement, predominantly of the anterior cingulate cortex (ACC) and orbitofrontal cortex (OFC; Boublay et al., 2016). Similarly, studies in subjects with MCI reported a link between apathy and hypoperfusion of the frontal, temporal, occipital lobes (Kazui et al., 2017) and inferior temporal and anterior cingulate atrophy (Guercio et al., 2015). Similar investigations were conducted also in non-AD dementias: in the behavioral variant of frontotemporal dementia (bvFTD), a close correlation was observed between disinhibition and atrophy of specific frontotemporal areas (ventromedial orbitofrontal, medial frontal, and anterior temporal lobe; Hornberger et al., 2011), while in Lewy body dementia (LBD), a dysfunction of both associative visual areas and limbic areas was found to be associated with the occurrence of hallucinations (Burghaus et al., 2012).

Given the variability of the evidence on BPSD so far available in the literature, this study aimed to expand the knowledge of the biological correlates of the neuropsychiatric symptoms, evaluating the impact of BPSD in different forms of cognitive impairment [MCI, AD, FTD, LBD, and Vascular Dementia (VD)], and searching for potential correlations between BPSD and CSF biomarkers and cortical visual rating scales.

## Materials and Methods

The study was designed and carried out at the IRCCS Mondino Foundation, with the collaboration of the University of Pavia, Italy. Recruitment started in June 2018 and was completed in February 2020.

All procedures contributing to this work comply with the ethical standards of the relevant national and institutional committees on human experimentation and with the Helsinki Declaration of 1975, as revised in 2008. Participants or their legal representatives provided written informed consent to all the diagnostic procedures included in this study. No participant received financial compensation.

### Participants

Participants were cognitively impaired patients undergoing diagnostic workup at the Behavioral Neurology Unit of the IRCCS Mondino Foundation. Inclusion criteria were: a diagnosis of MCI (amnestic or non-amnestic/single or multiple domains; Albert et al., 2011) or dementia; age between 50 and 90 years and an available informant with at least 10 h per week of contact with the patient. No limit of the severity of dementia was set, as long as the patient was able to perform a formal cognitive assessment. The vision and hearing acuity of patients were sufficient for compliance with testing procedures. Patients were excluded if they had a history of psychiatric disease or epilepsy, or any uncontrolled medical condition that could contribute to the subject’s cognitive impairment (e.g., nephropathy, liver disease, brain tumor, alcohol or drug abuse, normal pressure hydrocephalus). None of the patients were receiving medications for dementia, such as cholinesterase inhibitors or antipsychotic drugs, at the time of the diagnostic workup. Previously, 10 patients had taken antipsychotic drugs, and 11 had been on cholinesterase inhibitors.

### Study Design

The study was designed as a single-site cross-sectional study. Enrolled patients underwent complete clinical, neurological, and neuropsychological assessment, brain imaging (magnetic resonance imaging, MRI, or computed tomography CT), CSF collection (for the assay of Aβ42, total tau, and phospho-tau levels), and Neuropsychiatric Inventory (NPI) assessment. The neuropsychological examination included tests for global cognitive efficiency (Mini-Mental State Examination, MMSE), and for memory (Verbal Span, Digit Span, Corsi Test, 15 Item Memory Test, Story Recall Test, Rey Complex Figure delayed recall), logical and executive functioning (Raven’s Colored Matrices, Frontal Assessment Battery), attention (Trail Making Test A/B, Attentive Matrices, Stroop Test), language (Semantic and Phonemic fluency tests) and visual-spatial perception (Rey Complex Figure copy). NPI assessment was performed asking the caregiver to indicate *via* the screening questions whether the patient had experienced any domain-related neuropsychiatric symptom over the previous month. If the screening questions were validated, the caregiver was then asked to provide a domain rating for frequency, severity, and level of distress, and the total domain score was the product of the ratings for frequency and severity (Cummings et al., 1994). Four main NPI clusters (hyperactive behaviors, psychosis, affective behaviors, and apathy) were defined according to Aalten et al. (2008). “Hyperactive behaviors” cluster included agitation, euphoria, disinhibition, irritability, aberrant motor behavior, and night-time behavior disturbances; “psychosis” cluster included delusions and hallucinations; “affective behaviors” cluster included anxiety and depression; finally, “apathy” cluster included apathy and appetite/eating abnormalities.

Once the diagnostic workup was completed, patients were classified into two syndromic categories, MCI, and dementia, and the latter patients received an appropriate etiological diagnosis according to the most recent diagnostic criteria. MCI subjects were diagnosed according to NIA-AA criteria (Albert et al., 2011), and had clinical dementia rating (Morris, 1993) (CDR) = 0.5. Subjects with dementia received an etiological diagnosis of typical AD (Dubois et al., 2014), a behavioral variant of frontotemporal dementia (bvFTD; Rascovsky et al., 2011), Lewy body dementia (LBD; McKeith et al., 2017), or VD (Román et al., 1993), and had CDR ≧1. All patients with non-vascular dementia had a score <4 on the Modified Hachinski Ischemic Scale (Hachinski et al., 1975). Despite the advanced diagnostic workup including morphological and CSF biomarkers, 6 demented patients could not receive an etiological diagnosis with high confidence, and therefore were classified into a separate group, as not otherwise specified dementias (Dem NOS).

### Neuroimaging and CSF

MRI and CT scans were acquired at the Neuroradiology Unit of IRCCS Mondino Foundation. For this study, we analyzed 76 3D T1-weighted sequences acquired with Magnetom Skyra 3T (Siemens Healthcare), and 15 tomograms acquired with Somatome Perspective CT (Siemens Healthcare). MTA, PA, and the global cortical atrophy-frontal (GCA-F) scales were rated on 3D T1-weighted MR images, according to the original descriptions (Scheltens et al., 1992; Pasquier et al., 1996; Koedam et al., 2011), on both hemispheres. The MTA scale assesses the width of the choroid fissure and the temporal horn, as well as the height of the hippocampus; the PA scale assesses the width of the posterior cingulate- and parieto-occipital sulci, and the atrophy of the parietal lobe and precuneus; the GCA-F evaluates the severity of the atrophy of the frontal lobes. On CT images, only the MTA scale was rated. Visual ratings were collegially performed by two raters with more than 2 years of experience in the visual rating and over 900 neuroimages assessed from the LANE dataset (Cotta Ramusino et al., 2019). The raters were blind to diagnosis and CSF profile. Nine scans did not undergo visual rating due to the presence of artifacts or excess motion.

In 87 participants, a lumbar puncture was performed at the level of the L3/L4 or L4/L5 intervertebral space, according to the standard procedure used for patients with cognitive disorders in our clinic. CSF samples were centrifuged for 10 min at 1,800 *g* at 4°C within 3 h of collection. The samples were then divided into aliquots of 0.5 ml in polypropylene tubes and stored at −80°C. Measurement of CSF Aβ42, *t*-tau, and *p*-tau was performed using chemiluminescence enzyme immunoassay (Lumipulse G600II, Fujirebio); only for 14 participants, Aβ40 was available due to the relatively recent introduction of this assay in our laboratory. Biomarker profile was considered suggestive of AD pathology if Aβ42 < 599 pg/ml, *t*-tau > 404 pg/ml and *p*-tau > 56.5 pg/ml, or Aβ42/*t*-tau < 1.27, Aβ42/*p*-tau < 8.10 and Aβ42/Aβ40 < 0.069 (Lewczuk et al., 2018; Alcolea et al., 2019).

### Statistical Analysis

Shapiro-Wilk test was used to investigate the distribution normality of the different variables. Demographic and clinical characteristics among diagnostic groups were compared using ANOVA or Kruskal–Wallis test for continuous variables, and Chi-square test (*χ*^2^) or Fisher’s exact test for categorical variables. Differences in BPSD and syndromic clusters’ distribution among diagnostic groups were assessed with the Kruskal-Wallis test. Bonferroni correction was used to control for multiple comparisons in *post hoc* analyses performed through Dunn tests. Correlations between NPI scores and CSF biomarkers or visual atrophy brain scales were calculated using the Pearson coefficient. For non-parametric variables, confirmation was obtained using the Spearman coefficient. Differences in atrophy scores’ distribution between patients with or without any of BPSD (e.g., agitation vs. non-agitation) were assessed with Fisher’s exact tests. A beta regression model with backward elimination was used to evaluate the relations of the dependent variable, NPI total score, with the following baseline predictors: age, gender, education, MMSE, diagnosis, *t*-tau, Aβ42, left and right MTA, left and right PA, left and right GCA-F. The variable *p*-tau was removed after a first correlation analysis between the predictors. After the normalization of the dependent variable, the beta distribution was tested through a Kolmogorov-Smirnov test. Statistical computations were performed using R v. 3.5.3 (The R Foundation for Statistical Computing). Two-sided *p*-values <0.05 were considered to indicate significance. Due to the heterogeneous nature of the sample, *p*-values between 0.05 and 0.1 were also reported suggesting possible significance in further studies.

## Results

Demographic and clinical characteristics and biomarker measurements of the study population are shown in Table 1. Seventy-four patients were diagnosed with dementia (mean age, 74.5 ± 7.0 years; 60% female) and 26 with MCI (72.7 ± 6.5 years; 46%). The mean MMSE score was 17.3 ± 5.2 (range: 4.9–26.1) in patients with dementia and 26.8 ± 2.0 (range: 23–30) in subjects with MCI. In the dementia group, 48 patients were diagnosed as AD, seven as FTD, four as LBD, nine as VD, and six as Dem NOS. No significant difference was found among AD, non-AD, and MCI groups concerning age, gender, and education. As expected, the AD group showed lower Aβ42 levels, and higher *p*-tau and *t*-tau levels, compared to non-AD and MCI groups. No significant difference was found among the three groups with regard to atrophy scales scores.

**Table 1:** Sociodemographic features, cognitive status, and biomarker measures in the total sample and in three main diagnostic groups.

	Total sample (*N* = 100)	Dementia (*N* = 74)		MCI (*N* = 26)	*p*-value^a^
		AD (*N* = 48)	non-AD (*N* = 26)		
Age, mean (sd), years	74.1 (6.9)	74.4 (6.5)	74.8 (8.0)	72.7 (6.5)	0.31
Female, *N* (%)	44 (44)	30 (62.5)	14 (53.8)	12 (46.2)	0.45
Education, mean (sd), years	7.8 (3.6)	7.5 (3.8)	7.8 (4.1)	8.2 (2.6)	0.43
MMSE, mean (sd)^b^	19.9 (6.2)	16.7 (5.4)	18.6 (4.7)	26.8 (2.0)	<0.001
NPI tot, mean (sd)	32.6 (30.0)	33.8 (31.8)	38.0 (29.3)	25.0 (26.7)	0.33
CSF biomarkers, mean (sd), pg/ml					
Aβ_42_^b^	707.9 (360.2)	528.9 (230.9)	904.8 (394.0)	838.7 (376.5)	<0.001
*p*-tau^b^	72.5 (39.4)	95.7 (39.9)	44.6 (15.7)	57.4 (29.5)	<0.001
*t*-tau^b^	541.3 (351.8)	761.3 (360.3)	306.5 (117.2)	375.0 (235.3)	<0.001
MTA, N	91	44	23	24	
0 L/R	13/16	6/6	2/3	5/7	0.07/0.30
1 “	16/24	7/11	3/4	6/9	
2 “	36/28	16/15	8/7	12/6	
3 “	22/19	14/10	7/7	1/2	
4 “	4/4	1/2	3/2	0/0	
GCA, N	75	35	17	23	
0 L/R	18/18	7/7	2/2	9/9	0.24/0.30
1 “	27/28	13/14	6/6	8/8	
2 “	24/26	12/11	6/9	6/6	
3 “	6/3	3/3	3/0	0/0	
PA, N	76	36	17	23	
0 L/R	20/25	5/7	5/6	10/12	0.05/0.09
1 “	31/36	13/18	9/8	9/10	
2 “	21/11	14/7	3/3	4/1	
3 “	4/4	4/4	0/0	0/0	

*AD, Alzheimer’s disease; CSF, cerebrospinal fluid; GCA, global cortical atrophy; L, Left; MCI, mild cognitive impairment; MMSE, Mini-Mental State Examination; MTA, medial temporal atrophy; NPI, Neuropsychiatric Inventory; PA, posterior atrophy; R, right. ^a^Significance tests used were Fisher test for categorical variables and Kruskal–Wallis test for continuous variables. ^b^*Post hoc* pair-wise comparisons with Bonferroni correction of diagnostic groups: MMSE: MCI > AD and non-AD (*p* < 0.001); Aβ_1–42_: AD < non-AD and MCI (*p* < 0.001); *p*-tau: AD > non-AD and MCI (*p* < 0.001); *t*-tau: AD > non-AD and MCI (*p* < 0.001)*.

### Neuropsychiatric Symptoms Among Diagnostic Groups

**Figure 1:**
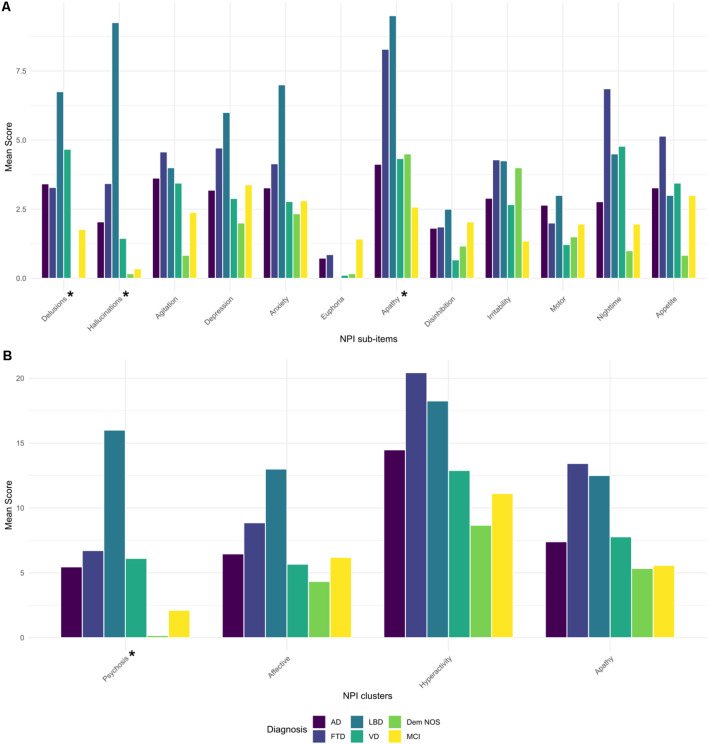
Barplot of mean scores of NPI sub-items **(A)** and NPI clusters **(B)** by diagnostic groups. AD, Alzheimer’s disease; FTD, frontotemporal dementia; Dem NOS, demented not otherwise specified; LBD, Lewy body dementia; MCI, mild cognitive impairment; NPI, Neuropsychiatric Inventory; VD, Vascular dementia. *Significance tests used were Kruskal–Wallis: Delusions (*p* < 0.05), Hallucinations (*p* < 0.001), Apathy (*p* < 0.01), Psychosis cluster (*p* < 0.05). *Post hoc* pair-wise comparisons with Bonferroni correction: Hallucinations: LBD > MCI and AD (*p* < 0.001 and 0.05, respectively), Psychosis cluster: LBD > MCI (*p* < 0.05).

NPI total score was not significantly different among diagnostic groups. Concerning the sub-items, delusions, hallucinations, and apathy were differently distributed among the diagnostic groups (*p* < 0.05, <0.001, and <0.01, respectively), as well as the psychosis cluster when NPI clusters were computed (*p* < 0.05; Figure 1). After *post hoc* analyses, hallucinations showed higher scores in LBD compared to MCI and AD patients (*p* < 0.001 and *p* < 0.05, respectively), and the psychosis cluster also displayed higher scores in LBD concerning MCI subjects (*p* < 0.05).

### Neuropsychiatric Symptoms and *t*-Tau

**Figure 2:**
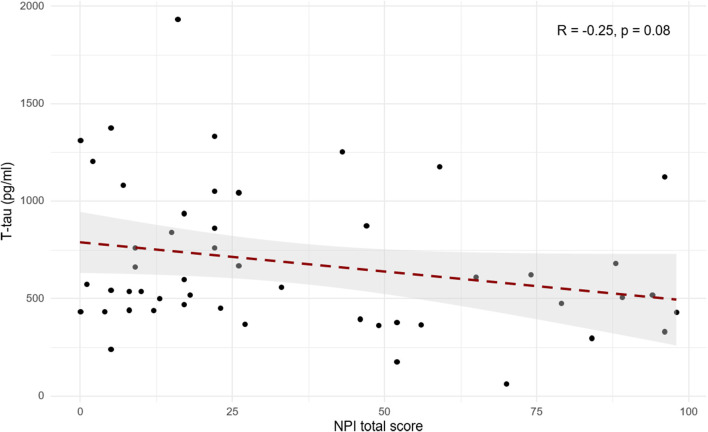
Scatterplot of Pearson’s correlation between cerebrospinal fluid (CSF) *t*-tau and NPI total score in patients with primary dementia (AD, FTD, and LBD).

When searching for correlations between NPI and *t*-tau levels in patients with primary dementia (AD, LBD, and FTD), we found a trend toward a negative correlation (*R* = −0.25, *p* = 0.08; Figure 2). Moreover, after backward elimination, the beta regression model indicated a significant association between NPI total score and *t*-tau (*p* < 0.01; Table 2). As far as each sub-item of NPI was concerned, a significantly negative correlation was found between nighttime disturbances and *t*-tau both in patients with primary dementia (*R* = −0.29, *p* < 0.05) and in the total sample (*R* = −0.22, *p* < 0.05).

**Table 2:** Beta regression model of association of Neuropsychiatric Inventory (NPI) total score and sociodemographic and biomarker variables, displaying predictors retained in the final model after backward elimination.

	Estimate	Std. error	*Z*-value	*p*-value = pr(>|z|)
(Intercept)	4.204	2.144	1.961	0.050
Age	−0.053	0.026	−2.018	**0.044**
Male gender	−1.661	0.444	−3.741	**0.0002**
Education	−0.099	0.051	−1.932	0.053
MMSE	0.036	0.030	1.198	0.231
*t*-tau	−0.002	0.001	−2.855	**0.004**
Aβ_42_	−0.001	0.001	−1.820	0.069
GCA L	−1.178	0.831	−1.418	0.156
GCA R	1.844	0.912	2.022	**0.043**

*GCA, global cortical atrophy; L, Left; MMSE, Mini-Mental State Examination; R, right*.

### Neuropsychiatric Symptoms and Brain Atrophy

When patients were dichotomized into two groups based on the presence or absence of a specific NPI domain (e.g., agitation positive vs. agitation negative), significantly higher GCA-F scores were found in patients with the following symptoms (Figure 4): delusions (*p* < 0.05, on both sides), hallucinations (*p* < 0.01 and *p* < 0.05, on left and right side, respectively) and apathy (*p* < 0.05, on both sides). No significant differences were found regarding MTA and PA scores.

**Figure 3:**
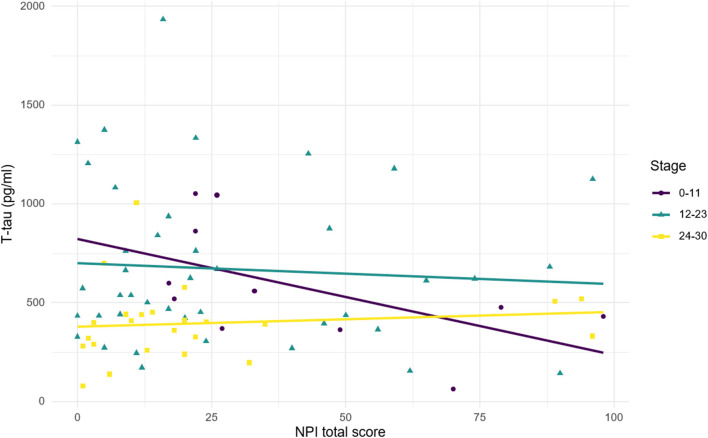
Relationship between CSF *t*-tau and NPI total score according to disease stages. Stage was defined according to MMSE score. 24–30, mild cognitive impairment; 12–23, mild-to-moderate dementia; 0–11, moderate-to-severe dementia. Correlations: stage 24–30: *R* = 0.11, *p* = 0.597; stage 12–23: *R* = −0.07, *p* = 0.657; stage 0–11: *R* = −0.55, *p* = 0.083.

**Figure 4:**
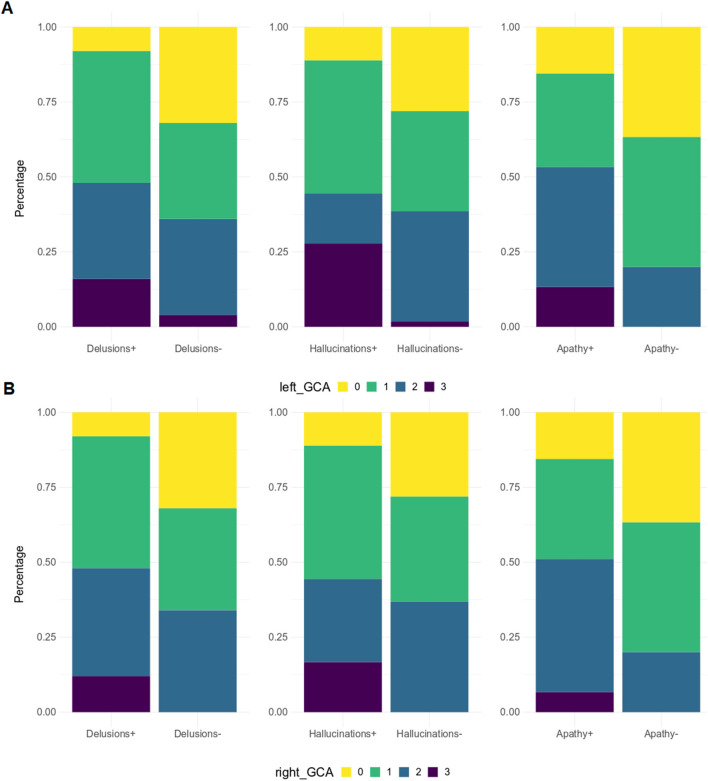
GCA-F score distribution by NPI sub-items. GCA-F, global cortical atrophy; NPI, Neuropsychiatric Inventory. GCA-F scores were reported only for NPI sub-items with significant comparative changes [panel **(A)** for the left hemisphere, and panel **(B)** for the right hemisphere]. Significance tests used was Fisher test: Delusions (*p* < 0.05), Hallucinations (*p* < 0.01), Apathy (*p* < 0.05).

Figure 3 illustrates the relationship between *t*-tau and NPI total score according to the stage of disease (scored with MMSE). A positive non-significant relationship was observed in MCI subjects, while a negative relationship was found in patients with dementia. Although the relationship is stronger in lower MMSE score, it shows a greater slope for higher MMSE score (MMSE = 12–23: *R* = −0.07, *p* = 0.657; MMSE = 0–11: *R* = −0.55, *p* = 0.083). The heterogeneity of the diagnostic groups included in the sample could partly explain the lack of significance observed in the correlations between *t*-tau and NPI total score (both in the whole sample and in the subgroups by MMSE range).

From correlation analyses, a positive correlation emerged between GCA-F scores and delusions (right: *p* < 0.05), agitation/aggression (left: *p* < 0.05), and psychosis cluster (right: *p* < 0.05). Conversely, nighttime disturbances were positively correlated with both GCA-F and MTA scores on both sides (left: *p* < 0.01; right: *p* < 0.05). Finally, following the correlation analyses, the beta regression model indicated a significant association between NPI total score and right GCA-F score (*p* < 0.05; Table 2).

## Discussion

This study investigated the distribution of neuropsychiatric symptoms and their CSF and neuroimaging correlate in a large sample of patients suffering from different dementing disorders, diagnosed with the most recent criteria. A significant prevalence of psychotic symptoms (hallucinations and delusions) was found in patients with LBD when compared to MCI or AD. The CSF biomarkers analysis showed a negative trend of *t*-tau when plotted with total NPI scores in patients with primary dementias, and a positive relationship in patients with MCI, drawing a parabolic trajectory across disease stages of increasing severity. However, the pathological heterogeneity of the sample and the small number of some etiological subgroups may have prevented the statistical significance of some analyses of correlation between *t*-tau and NPI score. Cortical atrophy in the frontal lobe was associated with the occurrence of delusions, hallucinations, and apathy, and correlated with the presence of agitation/aggression, while the atrophy of the medial temporal regions mainly correlated with night-time disturbances.

The high prevalence of hallucinations in our cohort of LBD patients is consistent with the most recent diagnostic criteria, which consider the hallucinations as one of the core clinical features, occurring in up to 80% of patients (McKeith et al., 2017) and predicting post-mortem Lewy pathology with 93% accuracy (Williams and Lees, 2005). This key symptom usually occurs in the form of well-structured visual hallucinations (VH), associated with different degrees of insight, even though tactile and auditory variants have been also reported (McKeith et al., 2017). Delusions are frequently reported in LBD, approximately in 49% of cases (Jellinger, 2012), the most frequent of them being represented by misidentification delusions, whose prevalence in LBD is higher than in AD (52% vs. 34%; Perini et al., 2016). The high frequency of psychotic symptoms observed in this study in LBD patients is therefore consistent with previous reports in the literature, supporting a higher prevalence of hallucinations and delusions in synucleinopathies, among which LBD, than in tauopathies, such as AD and FTD (Burghaus et al., 2012). Different functional and structural abnormalities have been suggested to underlie psychotic manifestations: parietal and occipital hypometabolism and frontal atrophy were reported in VH (Whitewell et al., 2007; Sanchez-Castaneda et al., 2010; Jellinger, 2012; Pezzoli et al., 2019), while hypoperfusion of frontal, limbic and paralimbic cortex was associated with misidentifications and delusions (Pernezckzy et al., 2008; Nagahama et al., 2010).

Interestingly, in our study, the negative trend observed between NPI total score and *t*-tau in patients with dementia was somewhat unexpected in front of the results of previous studies, which mostly report a positive correlation between *t*-tau and BPSD severity (Skogseth et al., 2008; Ramakers et al., 2013; Bloniecki et al., 2014; Babulal et al., 2016). Upon closer examination, these studies investigated populations with milder cognitive impairment than ours, including either healthy subjects (Babulal et al., 2016) or patients with MCI (Ramakers et al., 2013) or mild AD (MMSE = 24.2 ± 2.3; Skogseth et al., 2008). Conversely, the present study includes patients belonging to more advanced stages of the disease and with a more severe cognitive impairment, regardless of the etiological diagnosis (AD or non-AD; MMSE = 16.7 ± 5.4 and 18.6 ± 4.7, respectively). We, therefore, believe that *t*-tau decreases in advanced stages of dementia as a result of the decline of the neurodegenerative process involving the brain cortex, as also supported by longitudinal studies (Toledo et al., 2013). Unlike the above studies, Bloniecki and colleagues (Bloniecki et al., 2014) investigated the relationship between CSF biomarkers and BPSD in a cohort of cognitively more impaired patients (affected from mild-to-moderate AD, MMSE = 19.1 ± 4.2), and including a wider range of diagnosis (in addition to AD, also VD and AD mixed, MMSE = 20.2 ± 4.6). They found a positive correlation in the AD group alone, but with a decrease in the correlation coefficient (from 0.35 to 0.13) when the whole sample was analyzed (Bloniecki et al., 2014). The inclusion of non-AD dementias, usually characterized by lower *t*-tau values, could plausibly account for this finding. Similarly, the negative trend observed in our population of demented patients could partly originate from the presence of non-AD dementias (in particular, FTD and LBD), which may contribute to attenuating or reversing the correlation between *t*-tau and BPSD. On the other hand, the finding of a positive trend in our subjects with MCI appears to be consistent with the evidence present in the literature related to early disease stages. To summarize, after an initial increase directly related to the spread of the neurodegenerative process (Fagan et al., 2009), CSF *t*-tau levels may decline in advanced disease stages, as a result of the reduced number of neurons spared by atrophy and still likely to degenerate. Consistently with this interpretation, Mollenhauer et al. (2005) reported a decline in *t*-tau levels in AD patients in the advanced stage, while Isoe et al. (1996) described a biphasic curve with an increase in *t*-tau levels at the disease onset, followed by a progressive decline in the final stages.

In this study, we report for the first time that cortex visual rating can detect more severe atrophy involving the frontal lobe in patients with delusions, hallucinations, or apathy. The search for possible links between BPSD and cortical abnormalities is currently an active field of investigation. A recent study identified the reduction of volume of the frontal lobe (in particular, the anterior cingulate cortex and the middle frontal gyrus) as a significant predictor of the occurrence of BPSD, such as apathy, delusions, and hallucinations, in AD patients (Boublay et al., 2020). Previously, other studies had supported this link, reporting a positive correlation between frontal atrophy and the occurrence of apathy in AD patients (Apostolova et al., 2007; Bruen et al., 2008), and an association between frontal lobe dysfunction and decline in the initiative in AD, FTD and LBD patients, particularly in late disease stages (Robert et al., 2006; Peavy et al., 2013; Massimo et al., 2015). The involvement of frontal networks subserving motivation and reward mechanisms, including the anterior cingulate cortex, superior and middle frontal gyri, and basal ganglia, provides a possible explanation of how atrophy involving these areas may contribute to the loss of interest and the development of apathy (Levy and Dubois, 2006; Moretti and Signori, 2016). Several studies also reported a more frequent finding of frontal atrophy in demented patients with hallucinations. In these subjects, Sanchez-Castaneda et al. (2010) precisely described a more severe cortical atrophy involving the inferior frontal gyrus and the precuneus, while Heitz et al. (2015) reported functional impairment of both anterior and posterior cortical regions, including the anterior cingulate cortex, the orbitofrontal cortex, and the cuneus. In addition to other reports in the literature (Whitewell et al., 2007; Pezzoli et al., 2019), this evidence appears to be endorsed by the results of the present study. The above frontal areas are indeed included in neuronal circuits assigned to inhibitory control and the decision-making mechanisms; their deregulation could prevent the patient from inhibiting the production of internal images, thus representing the pathophysiological substrate for the development of hallucinations (Shine et al., 2011). Conversely, understanding the neurobiological bases of delusions is more challenging, as few studies addressed systematically this issue. As mentioned above, frontal atrophy is a frequently encountered finding, that suggests the involvement of many areas of the frontal lobe in the generation of delusions, with particular regard to the orbitofrontal, limbic, and paralimbic regions (Mentis et al., 1995; Geroldi et al., 2000; Mega et al., 2000; Sultzer et al., 2003; Bruen et al., 2008; Pernezckzy et al., 2008; Nagahama et al., 2010). Also, the positive correlation between delusions’ severity and right-sided GCA-F scores in our sample, confirms the suggested pivotal role of the right frontal lobe in controlling and structuring thought (Sultzer et al., 2003; Nakano et al., 2006; Devinsky, 2009). Based on this assumption, frontal cortical atrophy could promote the development of delusions through the loss of control functions aimed to supervise reality and to compare the internal experience with the outer world, leading to the consolidation of false beliefs (Richardson and Mallory, 1994). However, the meaning to attribute to the above findings is still largely speculative, mainly due to the heterogeneity of delusions’ presentation, which may reflect the impairment of multiple functional networks located in different areas of the brain.

Further links emerged from correlation analyses between cortical atrophy and BPSD: agitation positively correlated with left-sided GCA-F scores, whereas night-time disturbances positively correlated with GCA-F and MTA scores on both hemispheres. The few existing studies of neuroimaging investigating structural and functional correlates of agitation/aggression reported an involvement of the left frontotemporal region in AD patients, associated with a concurrent more severe burden of neurofibrillary tangles in the same region (Tekin et al., 2001; Trzepacz et al., 2013). These findings are consistent with a large body of neuropsychiatry literature describing a complex brain network of prefrontal, subcortical, and mesolimbic circuitry that mediates and regulates social behaviors, and of frontoinsular circuitry that plays a crucial role in the processing of more complex social emotions such as empathy, compassion, and fairness (Menon and Uddin, 2010). Agitation and aggression may therefore be due to the default of this frontotemporal network, leading to the loss of capacity to process and regulate behaviors properly (Trzepacz et al., 2013).

Sleep disorders are a particularly disabling aspect common to many forms of dementia, representing stressful conditions for patients and caregivers, and a major risk factor for early institutionalization. Micro-architectural sleep alterations, nocturnal sleep fragmentation, decrease in nocturnal sleep duration, diurnal napping, and even inversion of the sleep-wake cycle are the main disorders observed in patients with AD (Peter-Derex et al., 2015). In the present study, a positive correlation was found between night-time disorders and medial temporal atrophy; nonetheless, other structures involved in arousal regulation, as the hypothalamic suprachiasmatic nucleus (SCN), was previously correlated to sleep disruption (Lyketsos et al., 2007). It was proposed that MTA may induce dysfunction of the SCN and/or its downstream effector systems, and that, in turn, the fragmented and irregular rhythms of activity may aggravate the neuropathological process responsible for the MTA, according to a negative feed-forward cycle (Musiek et al., 2015; Van Someren et al., 2019).

## Limitations

The main limitation of this study is the heterogeneous composition of the population and the small size of some subgroups. The larger number of AD patients may have partly affected the correlation analyses between CSF or neuroimaging biomarkers and BPSD severity. Indeed, primary dementia, such as AD, FTD, and LBD, usually show different levels of tau and different cortical atrophy distribution, as a result of the underlying neuropathological process and its specific tropism. Moreover, some kinds of dementia are known to be characterized by specific BPSD, and these are purposely included in the diagnostic criteria (e.g., apathy in FTD, and hallucinations in LBD). The variable contribution of these nosological entities to the overall study population may have affected the percent presentation of BPSD, and may therefore have introduced a selection bias in the analyses performed on the whole sample. Finally, the small size of FTD and LBD groups may have been a limiting factor influencing the significance in the analyses according to sub-groups.

## Conclusions

This study provides a real-world overview of the most clinically relevant BPSD occurring in patients consecutively attending a memory clinic due to dementing conditions. The gathered evidence suggests that, in a future perspective, CSF biomarkers and visual rating scales for cortical atrophy could be hopefully included in a multidimensional evaluation of demented patients, aimed to predict prognosis and occurrence of BPSD. Longitudinal studies on wider and diagnosis-balanced cohorts of patients are however necessary to properly ascertain the actual predictive value of these biomarkers.

## Data Availability Statement

The raw data supporting the conclusions of this article will be made available by the authors, without undue reservation.

## Ethics Statement

Ethical review and approval was not required for the study on human participants in accordance with the local legislation and institutional requirements. The patients/participants provided their written informed consent to participate in this study.

## Author Contributions

MCR, GP, and AC: conceptualization. MCR, GP, MC, MP, DF, and LF: methodology; MCR, GP, BD, and EB: formal analysis and investigation. MCR, GP, and GV: writing—original draft preparation. MCR, GP, AC, DF, and EB: writing—review and editing. AC: resources and supervision. All authors contributed to the article and approved the submitted version.

## Conflict of Interest

The authors declare that the research was conducted in the absence of any commercial or financial relationships that could be construed as a potential conflict of interest.

## Abbreviations

AD: Alzheimer’s disease
BPSD: behavioral and psychological symptoms of dementia
CSF: cerebrospinal fluid
CT: computed tomography
FTD: frontotemporal dementia
GCA-F: global cortical atrophy-frontal lobe
LBD: Lewy body dementia
MCI: mild cognitive impairment
MRI: magnetic resonance imaging
MTA: medial temporal atrophy
NPI: Neuropsychiatric Inventory
PA: posterior atrophy.
